# The predictive value of transcranial duplex sonography for the clinical diagnosis in undiagnosed parkinsonian syndromes: comparison with SPECT scans

**DOI:** 10.1186/1471-2377-8-42

**Published:** 2008-11-09

**Authors:** Annemarie MM Vlaar, Tjerk de Nijs, Marinus JPG van Kroonenburgh, Werner H Mess, Ania Winogrodzka, Selma C Tromp, Wim EJ Weber

**Affiliations:** 1 Departments of neurology, university hospital Maastricht, The Netherlands; 2 Department of nuclear medicine, university hospital Maastricht, The Netherlands; 3 Department of clinical neurophysiology, university hospital Maastricht, The Netherlands; 4 Department of clinical neurophysiology, St. Antonius hospital, Nieuwegein, The Netherlands

## Abstract

**Background:**

Transcranial duplex sonography (TCD) of the substantia nigra has emerged as a promising, non-invasive tool to diagnose idiopathic Parkinson's disease (IPD). However, its diagnostic accuracy in patients with undefined parkinsonism remains to be determined.

In this study we determined the predictive value of TCD for the clinical diagnosis in undiagnosed parkinsonian syndromes. Additionally we compared the predictive value of TCD with that of presynaptic and postsynaptic single photon emission computer tomography (SPECT) scans.

**Methods:**

We studied 82 patients with an unclassified parkinsonian syndrome. All 82 patients were subjected to a TCD, 59 of them underwent a presynaptic SPECT scans and 32 underwent a postsynaptic SPECT scan.

We determined the diagnostic accuracy of TCD and SPECT scans in differentiating:

1) IPD patients from patients without nigrostriatal degeneration and 2) IPD patients from patients with atypical parkinsonian syndromes (APS).

To compare the diagnostic accuracy of TCD and SPECT scans, we used the clinical diagnosis after follow-up according to generally accepted clinical criteria as the gold standard. This clinical diagnosis was determined by a movement disorder specialist.

3) Finally, we ascertained the predictive value of the TCD for the SPECT result.

**Results:**

The clinical diagnoses after follow-up resulted in 51 cases of IPD, 7 patients with APS and 17 patients without nigrostriatal degeneration. In total 7 patients remained undiagnosed.

1) The accuracy of TCD, assessed by sensitivity and specificity, to differentiate IPD patients from patients without nigrostriatal degeneration was 50% and 82% respectively.

For the presynaptic SPECT scans sensitivity was 97% and specificity 100%.

2) In differentiating IPD patients from APS patients, the sensitivity and specificity of TCD was 50% and 43% respectively. For presynaptic SPECT scans this was 97% and 0%. For the postsynaptic SPECT scans the sensitivity was 75% and the specificity 81%.

3) The positive predictive value (PPV) of an abnormal TCD for an abnormal presynaptic SPECT scan was 88%.

**Conclusion:**

Presynaptic SPECT scanning has a higher predictive value for the clinical diagnosis than TCD. However, since the PPV of an abnormal TCD for parkinsonism with nigrostriatal degeneration is high, TCD might be used as screening tool, before ordering a presynaptic SPECT.

## Background

The diagnosis of idiopathic Parkinson's disease (IPD) is based on clinical criteria. When cardinal clinical signs and symptoms as bradykinesia, rigidity, and resting tremor are present, the diagnosis of IPD is straightforward [1]. However, the differentiation between IPD and other parkinsonian syndromes can be difficult, especially in the early stages of the disease.

Diseases which might resemble early-stage IPD are Multiple System Atrophy (MSA), Progressive Supranuclear Palsy (PSP), Diffuse Lewy Body Disease (DLBD), corticobasal degeneration (CBD), but also vascular parkinsonism (VP), drug induced parkinsonism (DIP) and essential tremor (ET). Clinicopathological studies show that 2–25% of the patients with IPD are classified incorrectly in the final stage of their disease, even by specialists in movement disorders [2-6]. Because the prognosis and medical treatment differ between various parkinsonian syndromes, an accurate and early diagnosis is essential for optimal treatment and counselling.

Single Photon Emission Computer Tomography (SPECT), with presynaptic and postsynaptic radiotracers, is widely used in the diagnostic work-up of patients with parkinsonian syndromes. Despite its widespread use, the exact diagnostic accuracy of this technique in parkinsonian syndromes remains somewhat controversial [7,8].

Becker and colleagues were first to report hyperechointensity of the substantia nigra (SN) in patients with IPD by using transcranial duplex sonography (TCD) [9]. Several studies found that this increased SN echointensity is present in 90% of IPD patients [9-12], presumably caused by a local increased iron content [13,14]. In healthy individuals this increased SN echointensity is found in only 9% [15]. Since Becker's first report, roughly 70 studies on the diagnostic accuracy of TCD in parkinsonian syndromes have been published. Most studies on TCD and IPD have been done on later-stage, well-diagnosed patients. However, this does not resemble the situation in clinical practice, where the neurologist would want to use TCD to reach a diagnosis in patients with a clinically unclear parkinsonisme.

In the present study we assessed the diagnostic accuracy of TCD of the SN (SN-TCD) in 82 consecutive patients with a recent, as yet unclassified, parkinsonian syndrome. We compared the diagnostic accuracy of SN-TCD with that of SPECT scan, which was done simultaneously in most of these patients. We also calculated the predictive value of the SN-TCD for the results of the SPECT scans.

## Methods

### Patients

We retrospectively studied 82 consecutive patients who presented at our outpatient clinic with a parkinsonism of yet unknown origin. All of these patients were subjected to a SN-TCD between 2004 – 2006 after giving their informed consent. The study was approved by the Institutional Review Board (committee for Medical Ethics) of the University Hospital Maastricht.

We initially studied 95 patients, of whom we had to exclude 13 (15%) with inconclusive SN-TCD's because of an inappropriate temporal bone window (i.e. insufficient to acquire a 2-dimensional image of the intracranial structures). This percentage of inconclusive results is within the normal range [15-17].

**Figure 1:**
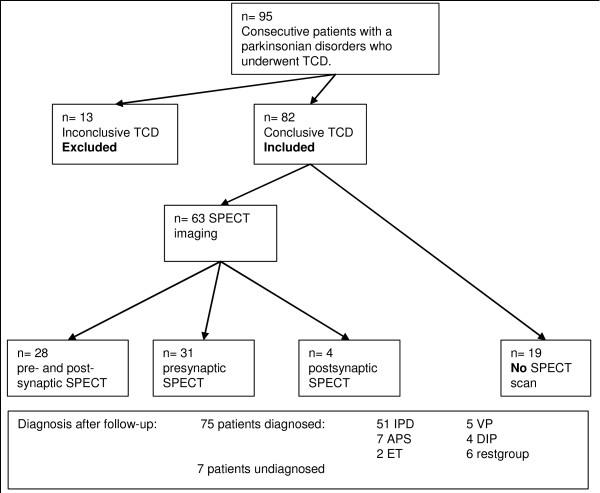
**Flowchart**. N: number of patients, TCD: transcranial duplex, SPECT single photon emission computer tomography, IPD: idiopathic Parkinson's disease, APS: atypical parkinsonian syndromes, ET: essential tremor, VP: vascular parkinsonism, DIP: drug induced parkinsonism, restgroup: two patients had spontaneous disappeared parkinsonism, 1 normal pressure hydrocephalus and 1 frontotemporal dementia.

Of the 82 patients, 59 underwent presynaptic-SPECT imaging and 32 postsynaptic SPECT imaging depending on the personal judgement of the treating specialist (Figure 1).

To reach a clinical diagnosis (the surrogate gold standard in this study) the patients were re-examined by an independent neurologist specialised in movement disorders, after an interval of at least six months.

A total of 52 patients (63%) gave consent to be re-examined (21 refused re-examination, 5 patients died, and 4 were lost to follow-up). However, the clinical records of the 30 patients that were not actually re-examined, were studied by the experts (WW, AW). They tried to reach a final clinical diagnosis for these patients, according to generally accepted clinical criteria.[1,3,18-21] If there was clinical doubt between different parkinsonian disorders a patient was called "*undiagnosed*".

### SN-TCD

We used a 2–4 MHz phased array transducer on a commercially available ultrasound system (SONOS 5500; Philips, Eindhoven, the Netherlands). The transducer was placed over the transtemporal bone window and focussed at a depth of 6–8 cm to allow for optimal image quality of the brainstem. Overall gain and the degree of compression were visually adapted to optimally depict an increased echointensity of the SN, if present. In practice a relatively high gain setting was used in combination with relatively little compression. This combination resulted in an almost homogeneously echolucent brainstem for the areas where no increase of the echointensity can be expected. The Raphe nuclei, the nucleus ruber and the SN (in case of a positive finding) on the other hand were easily distinguishable due to their increased echointensity. All other parameters like time gain compensation, post-processing and power settings were kept constant throughout the study.

Initially we performed also second harmonic imaging (insonation frequency 1.8 MHz). Yet it appeared, that with our ultrasound system we were able to visualize the brainstem only in a minority of patients. Since it is also well-known (personal conversation with Prof. D. Berg, Tübingen), that second harmonic imaging will lead to an increase of the hyperechointense area of the SN if compared to images based on the basal frequency content, we decided to use only primary images in this study.

A loop of 64 images was acquired by scanning the brainstem cranio-caudally through the pre-auricular bone window on the both sides. The images were stored digitally to allow for off-line analysis by a second sonographer. Both sonographers were blinded for the SPECT results and the clinical diagnoses. The echointensity of the SN was evaluated by ipsilateral insonation.

The SN echointensity can be estimated semi-quantitatively by manually encircling the area of hyperechointensity and having the ultrasound system measuring the area [10,15]. In our experience it is often difficult to accurately delineate the area of the SN hyperechointensity (if present), which would lead to inconsistent results. Therefore, we used a simplified and more practical method using the following qualitative scores: "*positive*", "*negative*", "*inconclusive*". Comparable qualitative scoring systems have also been used by other investigator groups [22-24].

The SN-TCD is scored "*positive*", when, at least on one side, the SN is clearly visible due to its increased echointensity as compared to the surrounding brainstem tissue. Additionally, the hyperechointense area has to correspond with the anatomy of the SN. Only if the hyperechointense area extends as an oblique and continuous band in the medial-anterior to lateral-posterior direction, we scored this as a "*positive*" finding. We could show that such an echo intensity increase always corresponded to an area larger than 0.2 cm^2 ^(unpublished data; currently under review).

If the image quality is sufficient to evaluate the echointensity of the SN, but the above mentioned criteria are not met, then the SN-TCD is scored "*negative*". This implies, that an SN-TCD is also called "*negative*" if there is a doubtful hyperechointensity. This could be either due to an atypical anatomical distribution or only a small area of hyperechointensity not extending throughout the whole brainstem in the horizontal plane as described above. An "*inconclusive*" SN-TCD is a measurement where visualization of the intracranial structures is not or only partly possible due to insufficient temporal bone windows on both sides.

### SPECT

In this study we used the presynaptic radiotracer FP-CIT (123I-Ioflupane, General Electrics Health, Eindhoven, The Netherlands) and the postsynaptic tracer IBZM (123I-iodobenzamide, General Electrics Health, Eindhoven, The Netherlands). Medication which could interfere with the radiotracer was stopped at least 5 half-life times before the SPECT was made. SPECT scans were performed with a triple head camera (MultiSPECT3, Siemens, Ohio, USA) equipped with high-resolution collimators. A semi-automatic template model programme was used to calculate the ratios between left striatal and right striatal and occipital regions, respectively. Total time of acquisition was 30 minutes (45 seconds per frame for 40 views per detector), zoom factor: 1.00 and the matrix size: 128 × 128. Filtered back-projection acquisition was performed. Images were filtered using a Butterworth filter with a cut-off value of 0.4–0.5 and an order of 5. The ratios were corrected using Alderson's brain phantom with known activities in the caudate nucleus and putamen.

A binding of two standard deviations below healthy controls was considered as abnormal (FP CIT 8.25, sd 1.85 for putamen and 7.76, sd 1.77 for caudate nucleus. IBZM for striatum 3.58, sd 0.18). The scans were analysed by a nuclear medicine specialist who was blinded for the clinical diagnosis and the SN-TCD results.

### Statistics

The diagnostic accuracy of SN-TCD, presynaptic and postsynaptic SPECT scintigraphy was determined by comparing their results to the surrogate gold standard: the clinical diagnosis after follow-up. Diagnostic accuracy is defined as the sensitivity, specificity, positive predictive value (PPV) and negative predictive value (NPV).

The accuracy was determined for: 1) IPD patients and patients without nigrostriatal degeneration (ET, DIP, VP, restgroup) and 2) IPD and APS patients.

And finally, we calculated the predictive value of SN-TCD for the presynaptic and postsynaptic SPECT result. SPSS (SPSS, Chicago, IL) was used for statistical analysis.

## Results

### Descriptives

The mean (standard deviation [SD]) age of the 82 patients was 69 (10) years, and the majority (65%) was male. The mean (SD) Hoehn and Yahr score was 2.4 (1.1). The follow-up duration after the SN-TCD was on average 17 (10) months.

In 75 (91%) of the 82 cases, it was possible for the movement disorders specialist to come to a definite clinical diagnosis. Seven cases remained undiagnosed after follow-up, because they did not fulfil the generally accepted clinical criteria [1,3,18-21]. For an overview of all the clinical diagnoses after follow-up see Table 1.

**Table 1:** Results of SN-TCD and presynaptic and postsynaptic SPECT imaging for each patient subgroup

clinical diagnosis after follow-up	SN-TCD number positive (= abnormal) of total	presynaptic SPECT number abnormal of total	postsynaptic SPECT number abnormal of total
IPD n = 51	25 of 51	37 of 38	4 of the 21
APS n = 7	4 of 7	6 of 6	3 of 4
• *MSA n *= *2*	*2 of 2*	*1 of 1*	*1 of 1*
• *PSP n *= *2*	*1 of 2*	*2 of 2*	*1 of 1*
• *CBD n *= *2*	*0 of 2*	*2 of 2*	*1 of 1*
• *DLBD n *= *1*	*1 of 1*	*1 of 1*	*0 of 1*
ET n = 2	0 of 2	0 of 2	-
VP n = 5	2 of 5	0 of 4	2 (visually 0) of 3
DIP n = 4	0 of 4	0 of 1	-
rest group n = 6^a^	1 of 6	0 of 4	0 of 1
undiagnosed n = 7^b^	3 of 7	3 of 4	1 of 3

n = 82	n = 82	N = 59	n = 32

n: number of patients, SN-TCD: transcranial duplex of the substantia nigra, SPECT: Single Photon Emission Computer tomography, IPD idiopathic Parkinson's disease, APS atypical parkinsonian syndromes, MSA multiple system atrophy, PSP progressive supranuclear paralysis, CBD cortical basal degeneration, DLBD diffuse Lewy body disease, ET essential tremor, VP vascular parkinsonism, DIP drug induced parkinsonism,

a: restgroup: two patients had spontaneous disappeared parkinsonism, 1 normal pressure hydrocephalus, 1 frontotemporal dementia.

b: undiagnosed: a patient was called undiagnosed if it was not possible to draw a conclusion on the final diagnosis according to generally accepted clinical criteria. [1,3,18-21]

### Accuracy of SN-TCD and pre- and post-synaptic SPECT in predicting the clinical diagnosis after follow-up

For the complete overview of the results of the SN-TCD, presynaptic and postsynaptic SPECT in each subgroup of patients, see Table 1.

The sensitivity of the SN in differentiating patients with IPD (n = 51) from patients with ET, VP, DIP or the rest group (n = 17) was 50%, while the specificity was 82%, the PPV 89% and the NPV 35%. For the presynaptic SPECT scans these values were respectively 97%, 100%, 100% and 92%.

SN-TCD could differentiate IPD patients (n = 51) from APS (n = 7) patients with a sensitivity of 50%, the specificity 43%; the PPV and the NPV were 86% and 10%, respectively. For the presynaptic SPECT scans, these values were 97%, 0%, 86% and 0% respectively, while for the postsynaptic SPECT the were 75%, 81%, 43% and 94%.

Of the 7 APS patients, 4 had a positive SN-TCD, while all 7 had an abnormal presynaptic SPECT. All 7 IPD and APS patients with an abnormal postsynaptic SPECT also had a significantly lowered presynaptic SPECT scan.

When the second sonographer (ST) assessed the SN-TCD, she obtained similar results in the vast majority of the patients (92%).

### Predictive value of SN-TCD for the results of the SPECT scan

59 patients underwent presynaptic SPECT imaging (see Table 2). In 33 patients (56%), the result of the SN-TCD was in line with the result of the presynaptic SPECT scan. The PPV of a positive SN-TCD for an abnormal SPECT was high: 88%. However, the NPV of a negative SN-TCD for a normal presynaptic SPECT was only 30%.

**Table 2:** Contingency of the 59 patients who underwent both SN-TCD and presynaptic SPECT imaging

Total number of patients n = 59	presynaptic SPECT abnormal	presynaptic SPECT normal
SN-TCD positive (= abnormal)	23^a^	3^c^
SN-TCD negative (= normal)	23^b^	10^d^

SN substantia nigra, TCD transcranial duplex, SPECT Single Photon Emission Computer Tomography

a: n = 23 19 PD, 3 APS, 1 undiagnosed

b: n = 23 18 PD, 3 APS (2 CBD, 1 PSP), 2 undiagnosed

c: n = 3 2 VP, 1 spontaneous disappeared parkinsonism

d: n = 10 1 PD, 2 VP, 2 ET, 1 DIP, 3 rest group, 1 undiagnosed

32 patients underwent postsynaptic SPECT imaging (see Table 3). In 14 (44%) of the 32 cases, the result of the SN-TCD was in line with the result of the postsynaptic SPECT scan. The PPV of a positive SN-TCD for an abnormal postsynaptic SPECT was 29% and the NPV 73%.

**Table 3:** Contingency of the 32 patients who underwent both SN-TCD and postsynaptic SPECT imaging

Total number of patient n = 32	postsynaptic SPECT abnormal	postsynaptic SPECT normal
SN-TCD positive (= abnormal)	6^a^	15^c^
SN-TCD negative (= normal)	3^b^	8^d^

SN substantia nigra, TCD transcranial duplex, SPECT Single Photon Emission Computer Tomography

a: IPD 2, APS 2, inconclusive 2

b: IPD 2, APS 1

c: IPD 13, APS 1, inconclusive 1

d: IPD 4, VP 3, 1 restgroup

## Discussion

Since the first report by Becker et al in 1995, SN hyperechogenicity in patients with IPD has been confirmed by many researchers [9,16,25-28]. These investigators have suggested that SN-TCD is an accurate instrument in discriminating IPD patients from normal healthy subjects, and from patients with other types of parkinsonism. However, most studies on TCD of the SN involve groups of well-diagnosed IPD patients, in the later stages of the disease. The diagnostic accuracy of SN-TCD in early-stage patient populations, with less well-defined clinical syndromes, thus remains to be determined. In an effort to do this, we did SN-TCD in 82 undiagnosed parkinsonian patients. As the gold standard for the patient's final diagnosis, we chose the clinical diagnosis after follow-up. This is, of course, not as good as post-mortem neuropathological analysis, but the best possible alternative at present.

In our study, the diagnostic accuracy of presynaptic SPECT is superior to that of SN-TCD. Especially the sensitivity and NPV in differentiating patients with parkinsonism *with *nigrostriatal degeneration (IPD, MSA, PSP, DLBD, CBD), from other causes of parkinsonism, are remarkably higher in presynaptic SPECT than in SN-TCD. However, the PPV of a positive SN-TCD for parkinsonism with nigrostriatal degeneration is high. This means that, although not all patients with nigrostriatal degeneration have a positive SN-TCD, a positive SN-TCD is a good predictor in diagnosing parkinsonism with nigrostriatal degeneration.

In our study, we also investigated the similarity in results between SN-TCD and presynaptic SPECT. We found a high PPV (88%) of a positive SN-TCD for an abnormal SPECT result, confirming other reports [29]. For clinical practice, this would imply that a positive SN-TCD in a patient with an early-stage, recently diagnosed parkinsonian syndrome, would reduce the added diagnostic value of a presynaptic SPECT.

A striking difference between ours and other studies, is the percentage of false-negative IPD patients diagnosed by SN-TCD. Our 50% is much higher than the 0–20% reported by other investigators [9,16,25-28]. There are several explanations for our lower sensitivity rate. Firstly, we used a broad spectrum of parkinsonian patients, which is representative for the diagnostic problem that one wants to solve. Other studies with similarly mixed patient groups also found a lower accuracy of the SN-TCD [22,23,30]. Secondly, our sonographers were blinded to the results of the clinical diagnosis. This might also have led to a decreased sensitivity of the SN-TCD, since we considered the SN-TCD negative in cases where we had doubts about the level of hyperechointensity. Thirdly, our patient population had a relatively large number of patients in the early stages of their disease. Although Berg et al. reported SN echointensity to be stable during follow-up, the question remains whether this is also the case in early-stage patient populations [31]. Fourthly, the quality of the ultrasound system is a non-neglectable variable, since in our pilot experiments, we found that the newest ultrasound systems will reveal hyperechointensity of the SN in more patients [32]. Finally, the TCD technique itself can provide an explanation. One needs considerable personal expertise to perform and interpret a TCD correctly. Since the pioneering research group in Tübingen has built up an enormous amount of expertise, it will be difficult for other groups to reproduce their excellent diagnostic results [25,32-37].

In summary, our study with early-stage parkinsonian patients, shows that the specificity and the PPV of SN-TCD for the final clinical diagnosis is just as high as it is for the SPECT scans. The sensitivity of the SN-TCD in our patient population is significantly lower than that of presynaptic SPECT scans. So in this population, the SN-TCD might be used as screening instrument: one could argue that in patients where the SN-TCD is compatible with parkinsonism with nigrostriatal degeneration, a presynaptic SPECT is no longer necessary in the diagnostic work-up. Applying this strategy, in our group of patients, 35% SPECT scans could have been omitted, resulting in a significant reduction in costs. Besides this, SN-TCD is also a more patient-friendly technique than SPECT scans.

## Conclusion

Our study with early-stage parkinsonian patients shows that the diagnostic accuracy of presynaptic SPECT scans is higher than that of SN-TCD. However, the specificity and the PPV of SN-TCD for the final clinical diagnosis, are almost as high as for presynaptic SPECT scans. So in this population, the SN-TCD might be used as screening instrument: patients with a SN-TCD compatible with parkinsonism with nigrostriatal degeneration would not need a presynaptic SPECT.

## Abbreviations

CBD: corticobasal degeneration; DIP: drug induced parkinsonism; DLBD: diffuse Lewy body disease; ET: essential tremor; FP-CIT: 123I-Ioflupane; IPD: idiopathic Parkinson's disease; IBZM: 123I-iodobenzamide; MSA: multiple system atrophy; NPV: negative predictive value; PPV: positive predictive value; PSP: progressive supranuclear palsy; SD: standard deviation; SN: substantia nigra; SN-TCD: transcranial duplex of the substantia nigra; SPECT: single photon emission computer tomography; TCD: transcranial duplex; VP: vascular parkinsonism.

## Competing interests

The authors declare that they have no competing interests.

## Authors' contributions

AMMV, TdeN, MJPGvanK, WHM, AW, SCT and WEJW were responsible for research project: A. Conception, B. Organization and C. Execution. AMMV, FGHK, WEJW were responsible for statistical analysis: A. Design, B. Execution and C. Review and Critique. AMMV, ST, WEJW were responsible for manuscript: A. Writing of the first draft. AMMV, TdeN, MJPGvanK, WHM, AW, SCT and WEJW were responsible for B. Review and Critique.

## Pre-publication history

The pre-publication history for this paper can be accessed here:


